# Supplementary material for: Feasibility of a Virtual Educational Programme for Behaviour Change in Cardiac Patients from a Low-Resource Setting
Source: Int J Environ Res Public Health. 2023 May 24;20(11):5934. doi: 10.3390/ijerph20115934 (PMC10252834; doi:10.3390/ijerph20115934)
Supplement: Supplementary file 1 [file ijerph-20-05934-s001.zip › ijerph-2301773-supplementary.pdf]

Scripts for interviews with patients and healthcare providers

Script - Questions to healthcare providers

1. What do you think worked well in the virtual educational program?
2. What do you think did not worked well in the virtual educational program?
3. What difficulties do you think the patients faced to participate in this virtual program?
4. What difficulties did you face in conducting the program?
5. What are some suggestions for implementation of this program in a large scale?

Script – Questions to patients

1. From 1 to 10 (1 being very unsatisfied and 10 very satisfied), how satisfied were you with the virtual education program? Why did you choose this grade?
2. What did you like most about the program?
3. What did you like least about the program?
4. From 1 to 10 (1 being very unsatisfied and 10 very satisfied), how satisfied were you with the following?
    a. The educational content
    b. The delivery of education
    c. The action plans and diaries
5. What were your reasons to participate in this virtual educational program?
6. Tell us about the program model and how useful the information received was and how effective the bi-weekly calls were?
7. Did your health behaviours change after the program participation?
8. What are some suggestions for the program in the future?
